# Monoclonal Antibodies Capable of Inhibiting Complement Downstream of C5 in Multiple Species

**DOI:** 10.3389/fimmu.2020.612402

**Published:** 2020-12-10

**Authors:** Wioleta M. Zelek, B. Paul Morgan

**Affiliations:** Systems Immunity Research Institute, Division of Infection and Immunity and Dementia Research Institute, School of Medicine, Cardiff University, Wales, United Kingdom

**Keywords:** complement, therapeutics, monoclonal antibody, C7, C5b-7, human, rat, mouse

## Abstract

Better understanding of roles of complement in pathology has fuelled an explosion of interest in complement-targeted therapeutics. The C5-blocking monoclonal antibody (mAb) eculizumab, the first of the new wave of complement blocking drugs, was FDA approved for treatment of Paroxysmal Nocturnal Hemoglobinuria in 2007; its expansion into other diseases has been slow and remains restricted to rare and ultra-rare diseases such as atypical hemolytic uremic syndrome. The success of eculizumab has provoked other Pharma to follow this well-trodden track and made C5 blockade the busiest area of complement drug development. C5 blockade inhibits generation of C5a and C5b, the former an anaphylatoxin, the latter the nidus for formation of the pro-inflammatory membrane attack complex. In order to use anti-complement drugs in common complement-driven diseases, more affordable and equally effective therapeutics are needed. To address this, we explored complement inhibition downstream of C5. Novel blocking mAbs targeting C7 and/or the C5b-7 complex were generated, identified using high throughput functional assays and specificity confirmed by immunochemical assays and surface plasmon resonance (SPR). Selected mAbs were tested in rodents to characterize pharmacokinetics, and therapeutic capacity. Administration of a mouse C7-selective mAb to wildtype mice, or a human C7 specific mAb to C7-deficient mice reconstituted with human C7, completely inhibited serum lytic activity for >48 h. The C5b-7 complex selective mAb 2H2, most active in rat serum, efficiently inhibited serum lytic activity *in vivo* for over a week from a single low dose (10 mg/kg); this mAb effectively blocked disease and protected muscle endplates from destruction in a rat myasthenia model. Targeting C7 and C7-containing terminal pathway intermediates is an innovative therapeutic approach, allowing lower drug dose and lower product cost, that will facilitate the expansion of complement therapeutics to common diseases.

## Introduction

The complement system comprises over 50 proteins, regulators and receptors circulating in plasma or on cells. Activation of the system by three distinct pathways, classical, lectin, and alternative, the latter comprising a common amplification loop, leads to formation of C3 convertases, followed by C5 convertases which cleave C5 into the potent chemotactic anaphylatoxin C5a, and C5b, the nidus for formation of the cytotoxic proinflammatory membrane attack complex (MAC). C5b while associated with the convertase, sequentially binds the plasma proteins C6 and C7, generating the C5b-7 complex that undergoes conformational change, triggering release from the convertase and exposing a labile hydrophobic membrane binding surface. The C5b-7 complex through its hydrophobic surface tightly binds membrane and sequentially recruits C8 and C9 to create the MAC, a transmembrane pore comprising one molecule each of C5b, C6, C7, and C8 and up to 18 copies of C9 that are recruited sequentially. The MAC pore allows metabolites and small proteins to leak out of the cell and water to flood into the cell due to osmotic pressure leading to lytic cell death (1–3).

Complement is critical to immune defense, providing recognition, tagging and elimination of bacteria and other foreign intruders and immune complexes; however, inappropriate activation of the system can lead to self-tissue and self-cell damage, driving disease. Hence, there is a need for therapies that block complement. For more than a decade, there was only one anti-complement drug in the clinic, the anti-C5 monoclonal antibody (mAb) eculizumab; this drug was FDA approved in 2007 for the ultra-rare hemolytic disease paroxysmal nocturnal hemoglobinuria (PNH), in 2011 for the ultra-rare renal disease atypical hemolytic uremic syndrome (aHUS), and for myasthenia gravis (MG) therapy in 2017 and in 2019 for the treatment of anti-aquaporin-4 (AQP4) antibody positive neuromyelitis optica spectrum disorder (NMOSD) in adults (1). At a list price of approximately $500,000 per patient per year, eculizumab, remains one of the most expensive therapies in the world. The drug must be given bi-weekly by intravenous infusion, 0.9 to 1.2 g/dose (1–5). These factors restrict progress toward therapy of more common complement-driven diseases. A step change is now needed to enable the use of anti-complement drugs in these conditions where there is considerable unmet need and many patients do not respond adequately to currently available agents. Anti-complement drugs for common conditions must be safer, cheaper and easier to administer.

Although the complement cascade can be targeted at many stages, anti-complement drugs in development are focused on very few targets, with agents mimicking eculizumab and targeting C5 or its breakdown products predominating. This is at least in part because inhibition of C5 has proven to be relatively low risk; the increased risk of Neisserial infections is managed by vaccination before treatment and prophylactic use of antibiotics. There are numerous agents in development that target C5, for example, crovalimab (SKY59), a C5 blocking mAb utilizing a pH-dependent recycling technology to increase drug half-life, and reduce the dose required to block C5 (in phase III clinical trials; (1, 6) https://clinicaltrials.gov/ct2/show/NCT04432584), and ravulizumab, the “next generation” eculizumab that also incorporates recycling technology enabling an increase in dosing interval to eight weeks. Ravulizumab has been FDA approved for PNH (2018) and aHUS (2019) (7).

Currently, only one drug targeting MAC downstream of C5 has progressed to clinical trials; AAVCAGsCD59 is a gene therapy agent in development for age-related macular degeneration (AMD); the agent is injected into the eye to locally express a non-anchored form of the MAC inhibitor CD59 (8). An anti-C6 mAb (CP010), developed by Complement Pharma and now partnered with Alexion, is in pre-clinical testing. (https://globalgenes.org/2018/06/12/alexion-and-complement-pharma-form-partnership-to-co-develop-complement-inhibitor-for-neurodegenerative-disorders/). In the wider literature there are a few preclinical reports describing targeting MAC beyond C5. An anti-C8 mAb was tested in hyperacute rejection (HAR) and cardiopulmonary bypass (CPB) rodent models; (9, 10) in HAR, mAb treatment protected hearts perfused with human serum while in CPB the mAb reduced platelet activation. A polyclonal antibody against C6 inhibited clinical symptoms in an experimental MG (EAMG) model in rats (11). Neither of these agents was advanced further. A very recent report described a monoclonal anti-C6 that inhibited hemolysis in human and rhesus monkey serum (12). These reports not only highlight the therapeutic potential of developing anti-terminal pathway drugs beyond C5, but also demonstrate the crucial role of MAC as a pathology driver.

Here we describe a panel of terminal pathway blocking mAbs, generated in C7-knockout (KO) mice hyper-immunized with human or rat C5b-7 and/or C7. The selected anti-human mAbs were equivalent or better inhibitors of human complement when compared to eculizumab in standard activity assays. Some of the mAbs showed cross-species activity when tested against human and rodent sera and the relevant mAbs efficiently inhibited complement activity *in vivo* in rodents. For one mAb, reactive against rat C5b-7, a single low dose inhibited complement for over a week in rats and blocked disease in the rat EAMG model.

## Materials and Methods

### Reagents and Sera

All chemicals, except where otherwise stated, were obtained from either Fisher Scientific UK (Loughborough, UK) or Sigma Aldrich (Gillingham, UK) and were of analytical grade. All tissue culture reagents and plastics were from Invitrogen Life Technologies (Paisley, UK). Sheep and guinea pig erythrocytes in Alsever’s solution were from TCS Biosciences (Claydon, UK). Eculizumab was kindly donated by Prof. David Kanavagh (Newcastle University, UK), and crovalimab by Roche Diagnostics (Basel, Switzerland). Cynomolgus monkey serum was purchased from Serlab (#S-118-D-24526, London, UK). Human and animal sera were prepared in house from freshly collected blood. For human, rabbit and rat, blood was clotted at room temperature (RT) for 1 h, then placed on ice for 2 h for clot retraction before centrifugation and harvesting of serum. For mouse, blood was placed on ice immediately after harvest and clotted for 2 h on ice before serum harvest. Sera were stored in aliquots at −80°C and not subjected to freeze–thaw cycles.

### Generation of mAbs

Monoclonal antibodies against C7/C5b-7 protein were generated by first establishing a line of C7 deficient mice. CRISP-generated heterozygous C7 KO mice (C57BL/6NJ-C7^em1(IMPC)J^/Mmjax) were purchased from Jackson Laboratories (Bar Harbour, Maine, USA) and back-crossed to obtain homozygous C7 deficient mice. The absence of C7 was confirmed by western blotting (WB) and hemolytic assays (data not shown). Wildtype (WT) and C7 KO mice were immunized with rat C7 and human C7/C5b-7 (both purified in-house) using standard schedules (13). The C7 KO mice were also used as a source of feeder macrophages during the cloning process. Immunized mice were screened using enzyme-linked immunosorbent assay (ELISA), mice with the highest titer response against the immunized proteins were selected and re-boosted before killing and harvesting of spleens. Plasma cells were harvested, fused with SP2 myeloma and aliquoted into 96-well plates. Hybridoma supernatants were screened using high-throughput hemolytic assay (described below) to identify blocking mAbs; supernatants with blocking activity were also screened for antibody responses by ELISA. Complement blocking mAb-secreting clones were sub-cloned by limiting dilution to monoclonality. Mouse mAbs were isotyped using IsoStrips (# 11493027001; Roche).

### Hemolytic Assays

The capacity of the mAbs to inhibit complement in human and animal sera was investigated by classical pathway (CP; CH50) hemolysis assay using antibody-sensitized sheep erythrocytes (ShEA); sheep blood was from TCS Bioscience and anti-ShE antiserum (#ORLC25, Siemens Amboceptor) was from Cruinn Diagnostics (Dublin, UK). ShEA were suspended in HEPES-buffered saline (HBS) containing Ca^2+^ and Mg^2+^ at 2% (vol:vol) (14). For measurement of activity in male mouse serum, ShEA were additionally incubated with mouse anti-rabbit IgG at 25 µg/ml (#3123; Invitrogen) for 30 min at 37°C before washing in HBS. A serial dilution series of each test mAb (100–0 µg/ml; 50 µl/well) was prepared in HBS and aliquoted in triplicate into a 96-well round-bottomed plate at 50 µl/well, then serum and 2% ShEA (50 µl/well of each) added. Serum dilutions for each species were selected in preliminary experiments to give near complete hemolysis in the absence of test mAb: 2.5%: normal human serum (NHS); 10%: normal Cynomolgus monkey serum (Cyno); 2.5% normal rat serum (NRS); 25%: normal rabbit serum (NRbS); 25%: normal male mouse serum (NMS) (using the double-sensitized cells as described above). Plates were incubated at 37°C for 30 min, centrifuged and hemoglobin in the supernatant was measured by absorbance at 405 nm. Percentage lysis was calculated according to: % Lysis = Absorbance (Abs) sample − Abs background)/(Abs max − Abs background) × 100%. GraphPad Prism was used for data analysis. Hybridoma supernatants were screened for blocking mAbs using the same assay but with neat tissue culture supernatant in place of the purified mAb.

Reactive lysis assays were used to identify mechanism of mAb inhibition. Guinea pig blood was from TCS; erythrocytes (GPE) at 2% in HBS (50 µl/well) were incubated sequentially with C5b6, C7, C8 and C9 (in house, affinity purified (15)), each for 10 min at 37°C, at doses titrated to give ~75% to 90% hemolysis in the absence of inhibitor. The concentrations (per well) of the purified components used were as follow; C5b6; 45 ng/ml, C7; 184 ng/ml, C8; 168 ng/ml, C9; 383 ng/ml. Molarities in nM; C5b6, 0.16; C7, 1.99; C8, 1.11; C9, 5.39 (Ratios: 1: 13: 10: 34). Serial dilutions (in triplicate) of the mAb (1–0 µg/ml) were made into HBS and added to the wells at different stages of MAC formation to determine the inhibition. Test and control mAb were added either prior to addition of C7 (human or rat), or prior to addition of C8/C9 and incubated for 30 min at 37°C. In some assay rat-EDTA serum was used as source of rat C8 and C9 to develop lysis of C5b-7 (human or rat C7) coated cells. Plates were centrifuged at 2000 rpm for 3 min at 4°C, supernatants removed to a flat-welled microtiter plate, absorbances measured spectrophometrically (A_405_nm) and % lysis calculated.

To reduce the need for large volumes of fresh mouse serum, add-back hemolytic assays were used for testing of lytic activity in mice treated with the mAb. NHS depleted of C7 (C7D) or C5 (C5D) was used as source of the rest of the complement proteins; mouse serum was added (1:2 v:v; mouse serum:C7D/C5D) to restore the relevant component. The serum mix was diluted to 10% in HBS then plated in a dilution series (50 µl/well; 10–0%) in HBS in a round bottom microwell plate; 50 µl 2% ShEA and 50 µl HBS were added (final concentrations of serum mix in wells: 3.3–0%), incubated for 30 min at 37°C. Plates were centrifuged, supernatants removed to a flat-welled microtiter plate and absorbance measured spectrophometrically (A_405_nm). % lysis was calculated.

### Characterization of Novel mAbs by ELISA and WB

Direct ELISA was used to test whether the new mAb bound C7 from different species. Sandwich ELISA were used to confirm C7 binders, to eliminate issues around denaturation by protein binding on plastic, and to test whether the mAbs competed for the same binding epitope. Standard curves were generated using in-house human or animal (rat, mouse, monkey) C7 proteins, immunoaffinity purified as previously described (15). In the direct ELISA, Maxisorp (Nunc, Loughborough, UK) 96-well plates were coated with C7 (0.5 µg/ml in bicarbonate buffer, pH 9.6) at 4°C overnight; wells were blocked (1 h) at 37°C with 2% bovine serum albumin (BSA) in phosphate-buffered saline (PBS), washed in PBS containing 0.05% Tween 20 (PBS-T). Dilutions of purified mAb, 1000–0 ng/ml (stock concentrations of all proteins used established using the BCA assay) in 0.2% BSA-PBS, were added in triplicate to wells coated with each of the antigens and incubated for 1 h at 37°C. Wells were washed with PBS-T then incubated (1 h, 37°C) with secondary antibody (donkey anti-mouse-horseradish peroxidase (HRP); Jackson ImmunoResearch, Ely, UK) for 1 h at 37°C. In the sandwich ELISA, Maxisorp plates were coated with mAb (2 µg/ml in bicarbonate buffer, pH 96) at 4°C overnight; wells were blocked (1 h at 37°C with 2% BSA-PBS) and washed in PBS-T. Standard curves were generated using in-house purified C7 protein serially diluted in 0.2% BSA-PBS, added in triplicate and incubated for 1 h at 37°C. Wells were washed with PBS-T then incubated (1 h, 37°C) with the paired HRP-labeled mAb (1 in 1000 dilution in PBS-T; Pierce, #31489). After washing, plates were developed using O-phenylenediamine dihydrochloride (OPD, Sigma FAST^™^; Sigma-Aldrich) and absorbance (492 nm) was measured. GraphPad Prism was used for data analysis.

To confirm specificity for C7 and the species reactivity, C7 protein (in house; 0.5 µg) or human or animal (mouse, rat, monkey) sera diluted 1:100 in PBS were placed in separate wells and resolved on 4–20% sodium dodecyl sulphate–polyacrylamide gel (SDS-PAGE) electrophoresis gels (#4561093; Biorad, Hemel Hempstead, UK) under reducing (R) and non-reducing (NR) conditions, then electrophoretically transferred onto 0.45 µm nitrocellulose membrane (GE Healthcare, Amersham, UK). After transfer, non-specific sites on the membrane were blocked with 5% BSA in PBS-T. After washing in PBS-T, membrane strips were incubated overnight at 4°C with individual test mAb (each at 1 µg/ml in 5% BSA PBS-T) or polyclonal (goat) anti-human C7 (2 µg/ml, CompTech, Tyler, TX; A224). After washing, bound test mAb were detected by incubation with donkey anti-mouse IgG-HRP (#715-035-150, Jackson ImmunoResearch) and polyclonal anti-C7 with rabbit anti-goat IgG HRP conjugate (#305-035-045; Jackson ImmunoResearch) at 1: 10000 in 5% BSA PBS-T. Blots were washed, developed with enhanced chemiluminescence (GE Healthcare) and visualized by autoradiography.

To characterize mAb binding to soluble terminal complement complexes (TCC), biotinylated mAbs (Pierce, #21327) were individually added to human or rat serum (100 µg/ml in 3 ml serum) and the mix activated *via* both classical and alternative pathways by incubation with Zymosan A (7 mg/ml; #21327, Pierce) and aggregated human IgG (1 mg/ml; in house) for 32 h at 37°C in a shaking water bath. The reaction was stopped by centrifugation at 2500 rpm for 15 min at 4°C and the supernatant (activated serum) collected. For analysis by WB, supernatant was mixed with 0.25 ml Avidin-coated beads (prepared by coupling avidin to HiTrap N-hydroxysuccinimide-activated beads; #17-0716-01, GE Healthcare, Little Chalfont, UK). The mixture was incubated for 1 hat RT while mixing gently, the beads washed five times in PBS by centrifugation (1000 rpm for 1 min) and the bound mAb-complex eluted by incubation (10 min at 100°C) in reducing or non-reducing SDS-PAGE running buffer. Supernatants were subjected to SDS-PAGE and WB as above. Blots were blocked with 5% BSA, washed, cut into strips and individual strips incubated with goat anti-C5, -C6, -C7, -C8 or -C9 antibodies (at 2 µg/ml, Comptech, Tyler, TX; A220, 223, 224, 225, 226) for 1 h, washed then incubated with horseradish peroxidase HRP-conjugated anti-goat immunoglobulin (1: 10 000 dilutions, #305-035-045; Jackson ImmunoResearch) for 1 h at RT with constant mixing. After washing, blots were developed as described above. For analysis by ELISA, supernatant was added to 96-well plates coated with Avidin (10 µg/ml), incubated 1 h at 37°C and blocked with 2% BSA (1 h at 37°C); after washing, terminal pathway components in the pull-down samples were detected with Goat anti-C5, C6, C7, C8 or C9 antibodies as above at 2 µg/ml diluted in 0.2% BSA PBS-T then developed with rabbit anti-Goat-HRP, #305-035-045; Jackson ImmunoResearch). Controls for each antibody comprised biotinylated mAb alone or incubated with C7. The assay was developed as described above.

Binding of the mAbs to pre-formed TCC was tested in ELISA; serum activated as above in the absence of mAbs (1 in 50 dilution in 0.2% BSA-PBS-T) was transferred to plates coated with aE11 anti-C9 neo-specific antibody (5 µg/ml, Hycult Biotech, # HM2167), incubated 1 h at 37°C and blocked with 2% BSA (1 h at 37°C); after washing, the new biotinylated mAb or as positive control, an in house mAb known to bind aE11-captured TCC (E2 anti-C8; 1 in 1000 dilution in 0.2% BSA-PBS-T) were added and incubated for 1 h at 37°C, after washing, Streptavidin-HRP (1 in 5000 dilution in 0.2% BSA-PBS-T, Fisher Scientific, # 21130) was added, plates incubated 1 h at 37°C and the assay developed as described above.

### SPR Analysis to Determine Test mAbs Binding Affinity to Human C7

The mAbs binding analyses were carried out on a Biacore T200 instrument (GE Healthcare); for mAb of isotype IgG, an antibody capture kit (GE Healthcare, # BR-1008-38) was used to immobilize the mAb on a CM5 sensor chip (GE Healthcare, #29-1496-03) as recommended by the manufacturer. mAb isotype IgM was immobilized on a Protein L Series S sensor chip (GE Healthcare #29-2051-38). mAb were flowed to saturate the surface, then C7, human, rat or mouse, diluted in EP-HBS (10 mM HEPES, pH 7.4, 150 mM NaCl, 0.005% surfactant P20) in the range 0 to 68 nM, flowed over the immobilized mAb. For kinetic analysis the flow rate was maintained at 30 µl/min, and data were collected at 25°C. Data from a reference cell were subtracted to control for bulk refractive index changes. The Rmax was kept low and the flow rate high to eliminate mass transfer. All reagents used were of high purity and polished by size exclusion chromatography immediately before use to ensure removal of any aggregates. Data were evaluated using Biacore Evaluation Software (GE Healthcare).

### *In Vivo* Testing of mAbs

To test *in vivo* mAb that blocked mouse complement in hemolysis assays, wild type (WT) mice (C57BL/6J, bred in house) were administered mAb by IP injection (1 mg in PBS, 40 mg/kg dose); controls included the blocking anti-mouse C5 mAb BB5.1 at the same dose. Blood was collected before mAb administration and at intervals up to 48 h after for measurement of hemolytic activity.

To test mAb that blocked human complement in hemolysis assays, C7 deficient mice (homozygous C57BL/6NJ-C7^em1(IMPC)J^/Mmjax, bred in house, n= 10) were injected intraperitoneally (IP) with human C7 (500 µg), then split into test and control groups (5 in each). One hour later, test group animals were injected subcutaneously (SC) with blocking mAb (1 mg in PBS, 40 mg/kg dose), while control group mice were injected with an irrelevant mAb at the same dose; blood was collected from all the animals just before the experiment, 1 h after C7 administration (immediately before giving the mAb) and at intervals after mAb injection up to 48 h for measurement of hemolytic activity.

To test mAb that blocked rat complement in hemolysis assays, Lewis rats (100–150 g; Charles River Laboratories, Edinburgh, UK) were injected IP with 10, 20 or 40 mg/kg dose of mAb (2 per group); blood was collected from all the animals just before the experiment, and at intervals post-injection over a time course of 7 days for measurement of hemolytic activity.

### Testing mAb in an Experimental Autoimmune Myasthenia Gravis (EAMG) Rat Model

A rat complement-blocking mAb was tested in the rat EAMG model. Lewis rats (100–150 g) were injected IP with anti-Acetylcholine receptor (AChR) mAb35 at 1 mg/kg in PBS as described previously (16–18). mAb35 binds the main immunogenic region of AChR, activating complement and damaging the neuromuscular junction endplates, causing severe damage to motor function. Animals were assessed hourly post-disease initiation as described previously. Clinical symptoms were assigned based on a standardized scale 0 to 5: 0, no disease; 1, reduced grip strength in front legs (can grip cage lid but cannot lift) and floppy tail; 2, loss of grip in front legs; 3, loss of grip and hind limb weakness and wasting; 4, loss of grip and hind limb paralysis; 5, moribund. mAb35-injected rats were split into two groups (n= 5 each), the test group received blocking mAb at 10 mg/kg SC (determined in the dosing experiment) at time zero, the control group received an irrelevant isotype control antibody (D1.3) at the same times, routes and doses. Blood was taken at intervals for hemolysis assays; all animals were sacrificed at 48 h post-induction.

Soleus muscles were harvested and frozen in OCT mounting medium for sectioning as described previously (16–18). Sections were fixed in ice-cold acetone for 15 min at −80°C and then blocked for 30 min in 10% horse serum/2% BSA. After washing in PBS, sections were stained overnight at 4°C with primary antibodies, C3/30 anti-C3b/iC3b mAb (in house) at 10 µg/ml, and rabbit anti-rat C9/MAC polyclonal IgG (in house) at 50 µg/ml, both in the block buffer. Anti-C3b/iC3b sections were washed and incubated for 15 min at RT with amplifier antibody goat anti-mouse (VectaFluor DyLight 488, # DK-2488; Vector Labs, Peterborough, UK). After washing, secondary antibody, horse anti-goat IgG–Alexa Fluor 488 (DyLight 488, # DK2488) for C3b/iC3b or goat anti-rabbit-FITC (#45002; Oxford Biomedical Research, Rochester Hills, MI, USA) for anti-C9/MAC were added as appropriate, together with α-bungarotoxin-TRITC (BtX) (labels AChR; Boitum, # 00012) at 0.5% and Hoechst stain 1: 10 000 dilution (# 62249; ThermoFisher), then incubated 40 min at RT in the dark. Sections were washed in PBS and mounted in VectorShield Vibrance (#H-1700-2; Vector Labs) before analysis using an Apotome fluorescence microscope (Zeiss Apotome Axio Observer microscope, Carl Zeiss Microscopy, Cambridge, UK). Ten fields were captured from comparable regions of muscle in each sample at the same exposure and magnification (×20) and the number of BuTx-reactive endplates in each section was measured using density slicing in an image analysis system (ImageJ, University of Wisconsin-Maddison, Maddison, WI, USA). For co-localization of complement activation products, sections were additionally imaged on a Zeiss confocal microscope (Zeiss LSM800 confocal laser scanning microscope).

### Statistical Analysis

All statistical analyses were performed using GraphPad Prism software (v. 5.0, San Diego, California). Statistical significance between two groups was obtained using the unpaired t-test and for multiple groups using one-way ANOVA after testing for normality. For all analyses, p<0.05 was considered significant. Error bars in all figures represent mean ± standard error of triplicates (unless otherwise stated. The SPR analysis was performed in an automated manner using T200 Biacore Evaluation Software version 2 (GE Healthcare).

## Results

### The Novel Blocking mAb Work Across Species and Block Hemolysis by Binding C7 and/or the C5b-7 Complex

**Figure 1:**
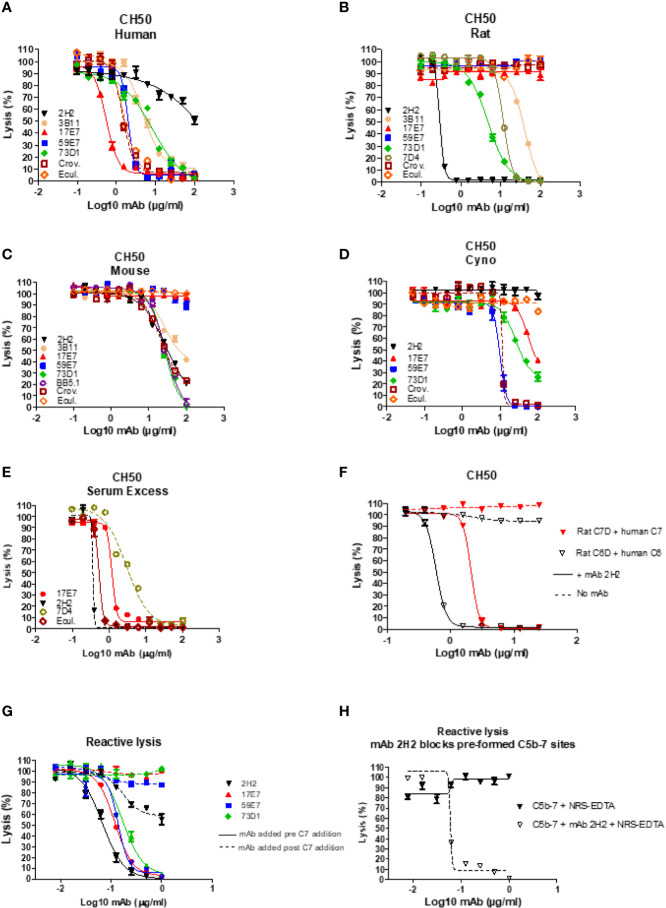
Functional assays of C7-blocking mAbs. **(A–D)** Anti-C7 mAb were tested for blocking activity in classical pathway (CP) hemolysis (CH50) assays across species. Sera tested were human **(A)**, rat **(B)**, mouse **(C)** and cynomolgus monkey (Cyno) **(D)**. Anti-C5 mAb crovalimab (Crov), eculizumab (Ecul), BB5.1 and 7D4 were used as comparators. Test and control mAb were titrated in range 0–100 µg/ml. **(E)** Serum excess assay using NHS or NRS concentration 10-fold that used in the standard CP assay; solid lines are NHS, dashed lines are NRS. **(F)** human C6D and C7D were reconstituted with purified human C6 or C7 (dashed lines) respectively and the capacity of mAb 2H2 to inhibit hemolysis tested. **(G, H)** Reactive lysis assays using guinea pig erythrocytes (GpE) as target; for mAb 17E7, 59E7, and 73D1, purified human complement proteins (C5b6, C7, C8 and C9) were used; for mAb 2H2 and 73D1 human C5b6 and rat C7 were used with normal rat serum (NRS) as the source of rodent C8 and C9. The mAb were either added to GpE-C5b6 before or after C7 addition **(G)**; solid and dotted line respectively. mAb 2H2 was added to washed GPE- C5b-7_(rat)_ prior to addition of NRS as source of C8 and C9 **(H)**. All experiments were repeated three times with the same results. The error bars are standard errors of triplicates.

In total, 15 fusions were performed; ~15,000 hybridoma clones were generated and screened, 7 confirmed to be inhibitory, and five of these, 2H2 (IgG2b, κ), 3B11 (IgM, κ), 17E7 (IgG2a, κ), 59E7 (IgG2b, κ), and 73D1 (IgG2a, κ), chosen for full characterization based upon the capacity of clone supernatants to cause inhibition of CP hemolysis of ShEA by NHS, NRS or NMS. All selected mAbs except mAb 17E7 were generated in C7 KO mice; 17E7 was produced in a WT mouse. All mAbs except 3B11 were purified using protein G chromatography, mAb 3B11, an IgM mAb, was purified using ammonium sulphate precipitation. The purified mAbs were tested in hemolysis assays with different species sera (**Figures 1A–D**). Anti-C5 blocking mAbs were used as positive controls; commercial mAbs eculizumab and crovalimab for NHS, BB5.1 for NMS and in house mAb 7D4 for NRS (18, 19). As expected from the selection process, each of the selected mAbs efficiently inhibited CP hemolysis in one or more species sera; In NHS, mAbs 17E7, 59E7, 3B11 and 73D1 all inhibited in that order of efficiency; 2H2 inhibited weakly (**Figure 1A**). In NMkS 59E7, 73D1, and 17E7 inhibited in that order of efficiency; no inhibition was observed with 2H2 (**Figure 1D**). In NRS, 2H2 was an exceptionally strong inhibitor, at least ten-fold better than other mAbs in the assay; 73D1 and 3B11 also inhibited NRS in that order of efficiency, but 17E7, 59E7, and the commercial anti-human C5 mAb had no inhibitory activity in NRS (**Figure 1B**). In NMS, mAbs 73D1, 2H2, and 3B11 inhibited in that order of efficiency, but 17E7, 59E7, and the C5-blocking controls 7D4 and eculizumab had no inhibitory activity in NMS (**Figure 1C**). The 73D1 inhibition profile closely matched that of BB5.1, the blocking anti-C5 mAb used as positive control in this assay; crovalimab also inhibited NMS as previously reported (**Figure 1C**) **(** 19). None of the new mAb inhibited in NRbS (negative data not shown). The cross-species inhibitory activities of the mAbs are summarized in **Table 1** **;** the calculated 50% complement inhibitory doses and hemolytic units (HU) of all mAbs in the different sera are shown. Serum excess assays (25% serum;10-fold serum dose compared to titration assays above) were used to test mAb 17E7 and 2H2 in conditions that better reflect those prevailing in whole blood (**Figures 1E, F**), confirming that these mAbs are efficient complement inhibitors in human and rat serum respectively.

**Table 1:** Summary of novel anti-C7 and control anti-C5 blocking mAb tested.

Antibody	Isotype	Species	Hemolytic Units (HU)	50% Inhibitory Dose (ng/ml)	Reactivity
**mAb anti-C7**					
17E7	IgG2a,κ	Human	55.1	181.6	Strong
		Monkey	1.9	530.3	Strong
59E7	IgG2b,κ	Human	47.7	209.7	Strong
		Monkey	10.1	98.7	Strong
3B11	IgM,κ	Human	19.9	501.9	Strong
		Rat	11.9	839.5	Moderate
2H2	IgG2b,κ	Human	1.0	9977.0	Weak
		Rat	264.8	33.9	Strong
		Mouse	2.7	3715.4	Weak
73D1	IgG2a,κ	Human	17.2	581.8	Strong
		Monkey	3.8	261.1	Strong
		Rat	19.8	505.5	Strong
		Mouse	13.9	720.3	Strong
**mAb anti-C5 controls**					
7D4	IgG2b,κ	Human	69.3	144.2	Strong
		Rat	9.5	1054.4	Moderate
BB5.1	IgG1,κ	Mouse	16.3	613.6	Strong
Crovalimab	IgG1,κ	Human	49.9	212.3	Strong
		Monkey	8.6	115.5	Strong
		Mouse	1.4	1862.1	Weak
Eculizumab	IgG2/4,κ	Human	47.1	181.6	Strong

Inhibitory dose: <750, strong; 750–1500, moderate; >1500, weak.

To identify the precise mechanism of complement inhibition by the novel mAbs, reactive lysis assays were used. GPE were first incubated with C5b6; blocking mAbs (17E7, 59E7, 73D1, 2H2) at various doses (0–1 µg/ml) added either before or after C7 addition, followed by C8, C9. All mAb except 2H2 were tested with purified human proteins; for mAb 2H2, purified rat C7 and EDTA-NRS as a source of rat C8 and C9 were used with human C5b6. All the 22 tested mAbs showed strong inhibition when added to GPE-C5b6 before C7 was added. When added after C7, mAb 17E7, 59E7, and 73D1 all showed no inhibition of lysis (**Figure 1G**). To further test the mode of inhibition of mAb 17E7 the mAb was either pre-incubated with C7 prior to addition to GPE-C5b6 or added simultaneously with C7; inhibition of lysis was essentially the same with or without the pre-incubation step, suggesting that the mAb efficiently captures fluid-phase C7 to prevent formation of an active C5b-7 complex (data not shown).

In contrast to the other mAb, the rat-selective mAb 2H2 caused a dose-dependent inhibition of lysis even when added to pre-formed GPE-C5b-7, implying that this mAb had a distinct mechanism of inhibition compared to the other mAb, working at least in part by binding and blocking C5b-7 thus preventing C8 binding to the complex (**Figure 1H**). When tested in a reactive lysis system using human C5b6, rat C7 and either human C8/C9 or NRS as a source of C8/C9, mAb 22H2 effectively blocked lysis when incubated with pre-formed GPE-C5b-7_(rat)_ and washed to remove free mAb prior to addition of either human C8/C9 or NRS, confirming the findings with human C7 above and demonstrating that the species source of C8/C9 (human or rat) did not impact the effect (**Figure 1H**). To further test species specificity of mAb 2H2, rat C6D was reconstituted with human C6 and rat C7D with human C7; in each case, 2H2 strongly inhibited lysis in a dose-dependent manner, implying that this mAb is selective for the C5b-7 complex (**Figure 1F**).

### The Novel mAbs Bind Native C7 and C7 in the TCC Complex in Serum

**Figure 2:**
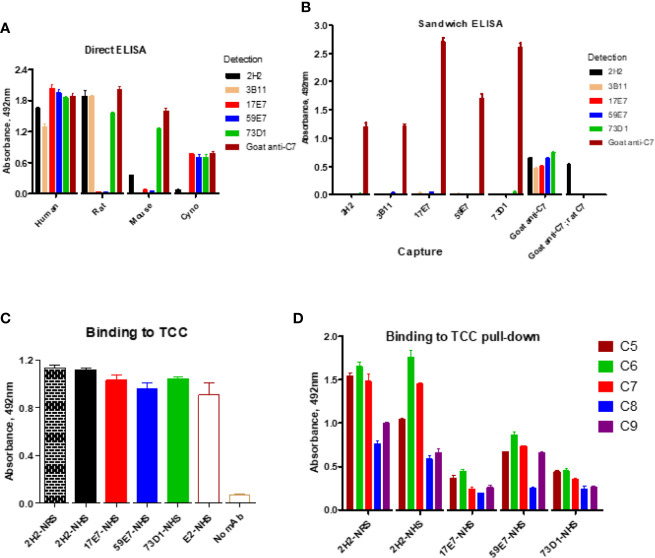
Direct and sandwich ELISA to determine mAb binding to human, monkey, rat and mouse C7. **(A)** Direct ELISA: plates were coated with human, monkey (cyno), rat or mouse C7; mAbs were tested in a dilution series (0–10 µg/ml). The graph shows representative binding with the 10 µg/ml capture. **(B)** Sandwich ELISA: the new mAbs were paired as capture and detect with human C7 used in a dilution series (0–5 µg/ml). The graph shows representative binding with 5 µg/ml C7. As a positive control, polyclonal anti-C7 (goat anti-C7) was used as capture with each of the mAb as detect; rat C7 captured on goat anti-C7 (goat anti-C7; rat C7) was also tested with the mAb as detect. **(C)** Sandwich ELISA to detect pre-formed TCC in activated NHS; TCC in activated NHS (and NRS for 2H2) was captured on aE11 anti-C9 and the novel mAbs and the positive control E2 anti-C8 mAb used to detect the TCC complex captured. **(D)** TCC complexes generated in NHS (or NRS for 2H2) in the presence of each of the new mAb (biotinylated) were captured on avidin-coated plates, then polyclonal antibodies against each of the terminal complement proteins used to test the presence of the respective components in the complex. The error bars are standard errors of triplicates. All experiments were repeated three times with the same results.

The direct ELISA showed that all the selected new mAbs recognized human C7; mAb 2H2, 3B11, and 73D1 also bound rat C7, while mAb 73D1 bound mouse C7. mAb 17E7 and 73D1 were strongly cross-reactive with non-human primate (cynomolgus) C7 (**Figure 2A** **;** **Table 1**). In competitive sandwich ELISA, C7 was not detected with any mAb pair suggesting that they compete for similar epitopes on C7; all mAb worked in sandwich ELISA with goat anti-C7 as either capture or detect (**Figure 2B**). mAb 2H2 when used as capture and goat anti-C7 as detection also detected rat C7, demonstrating that the mAb recognizes C7 from both species.

**Figure 3:**
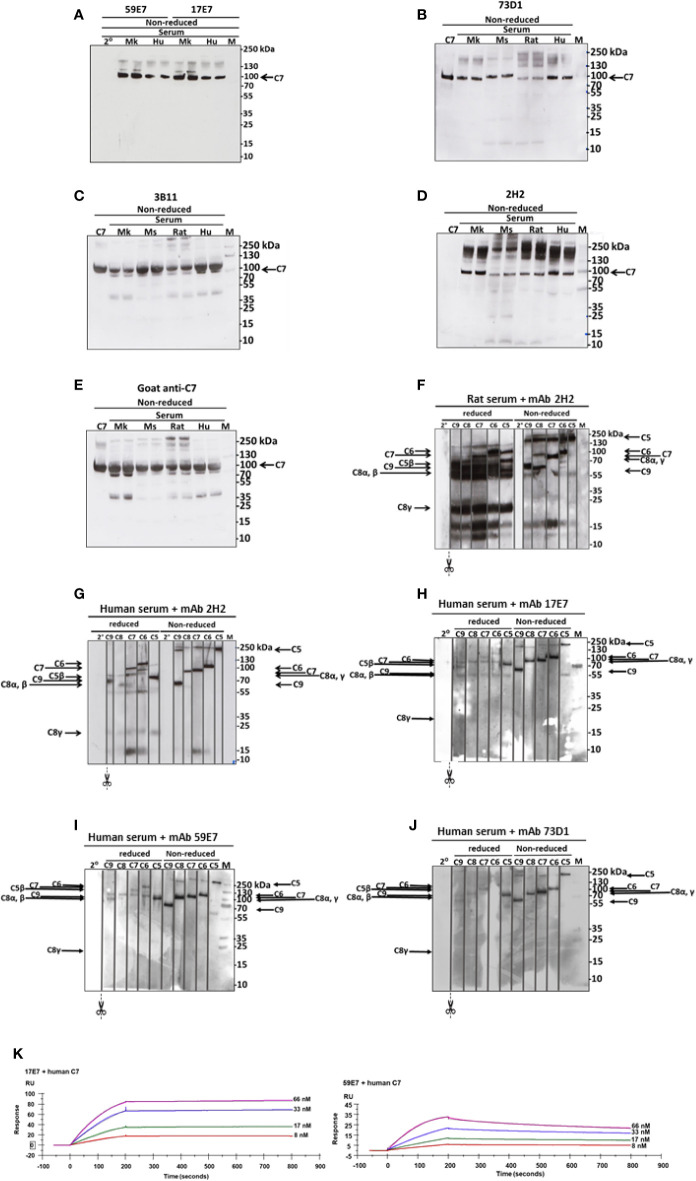
Western blotting to detect C7 binding in serum and in TCC. **(A)** The human-specific mAbs 59E7 and 17E7 were used to probe WB of NHS (Hu) and Cynomolgus monkey (Mk) serum under non-reducing (NR) conditions. Secondary only control was included (2°). **(B–E)** The cross-species reactive mAbs 73D1 **(B)**, 3B11 **(C)** and 2H2 **(D)** and as control the polyclonal goat anti-C7 **(E)** were used to probe WB of NHS (Hu), monkey (Mk), mouse (Mo) and rat sera; purified human C7 was used as standard. All sera were run in duplicate. Polyclon3B11 **(C)**, 2H2 **(D)**, and positive control goat anti-C7 **(E)**. Results are representative of three independent experiments. M; protein molecular weight marker. **(F–J)** The novel mAbs were used to pull down complexes from activated serum; these were then run on WB under non-reduced and reduced conditions and probed with polyclonal antibodies against each of the terminal complement proteins. mAb 2H2 was used in rat **(F)** and human **(G)** serum; the other mAbs in human serum only **(H–J)**. The blots were cut into strips prior to probing to detect the individual terminal pathway proteins. Molecular weights used were: NR: C5, 190 kDa; C6, 105 kDa; C7, 95 kDa, C8αγ; 70 kDa; C9, 65 kDa. R: C6, 110 kDa; C7, 95 kDa; C5β, 75 kDa; C9, 70 kDa; C8α/β, 65 kDa; C8γ, 22 kDa. Results are representative of at least three analyses. M; protein molecular weight marker, 2°; secondary antibody. **(K)** The novel mAbs 17E7, 59E7 and 73D1 were separately immobilized on mouse IgG capture sensor chips (GE Healthcare, # BR-1008-38) and mAb 3B11 (IgM) on protein L Series S sensor chip (GE Healthcare #29-2051-38) at approximately 60 RU. Human, rat or mouse C7 was flowed in HEPES-buffered saline (HBS) in a dilution range of 66 to 8 nM and interactions with the immobilized mAbs were analyzed. Sensorgrams were collected and KDs were calculated using the Langmuir 1: 1 binding model with RI values set to zero. Representative sensorgrams for 17E7 and 59E7 binding of human C7 are shown with raw data in colored lines and fitted data in dotted line (average of 3); all binding data and analyses are included in **Table 2**. The sensorgrams of clones 3B11 and 73D1 are included in Supplementary. The SPR analysis was performed in an automated manner using T200 Biacore Evaluation Software version 2 (GE Healthcare).

WB was used to confirm binding of mAbs to C7. The human-specific mAb 59E7 and 17E7 detected C7 in human and cynomolgus monkey serum under NR conditions (**Figure 3A**); these mAb did not detect C7 in other species sera, confirming the ELISA results above. The species cross-reactive mAb 73D1 and 3B11 specifically detected C7 in human, rat, mouse and cynomolgus monkey sera under NR conditions (**Figures 3B, C**). None of the mAb detected C7 in sera under R conditions (negative data not shown). mAb 2H2 did not bind the human C7 standard, detected by all other mAbs; it did weakly detect C7 in all sera tested under NR conditions but also identified multiple high molecular weight bands in the MW range 160 to 260 kDa that may represent C7 aggregates or terminal pathway complexes in serum (**Figure 3D**). To further explore the nature of these, pull-down assay from rat or human serum activated with zymosan and aggregated IgG in the presence of biotinylated 2H2 was performed; remarkably, mAb 2H2 pulled down all the terminal pathway proteins from both rat and human serum, indicating that it bound the fluid phase TCC when present during activation (**Figures 3F, G**). Pull-downs from activated human serum performed as above but using biotinylated mAb 17E7, 59E7, and 73D1, showed that each of the antibodies also pulled down all the terminal pathway proteins C5b–C9, demonstrating capacity of each of these mAbs to bind C7 in the forming TCC (**Figures 3H–J**).

Binding of the novel mAbs to TCC when present during serum activation was confirmed in sandwich ELISA on pull-downs from rat or human serum using the mAbs as above. All terminal pathway proteins: C5b, C6, C7, C8, and C9 were detected in the pull-downs; the strongest signals were observed with C5b, C6, and C7 proteins (**Figure 2D**). All the new mAb also bound the pre-formed TCC (captured on aE11 anti-C9 neospecific antibody) in activated human serum, giving signals at similar levels to the positive control E2 anti-C8 antibody, confirming that they bind C7 in the pre-formed TCC (**Figure 2C**). The aE11 mAb also captured TCC from activated rat serum and this was detected using the rat C7-reactive mAb 2H2 (**Figure 2C**). Taken together, the data show that each of the mAb recognize C7 in the TCC both when present in the fluid phase during TCC generation, and when added post-activation to the preformed complex.

SPR analysis on immobilized antibody with human or rat C7 flowed over confirmed binding of the novel mAbs to human and/or rat C7. The measured kinetics/affinity are summarized in **Table 2**. The mAb 17E7 and 59E7 showed very strong binding to human C7 in SPR analyses (KD = 1.02 × 10^−9^, 9.31 × 10^−10^ respectively) (**Figure 3K**) with negligible off rates, suggesting that 17E7 and 59E7 are promising candidates for human therapeutics. Binding of mAb 3B11 and 73D1 to human or rat C7 was relatively weak (human KD = 2.30 × 10^−7^ and 5.55 × 10^−8^; rat KD =1.93 × 10^−7^ and 8.17 × 10^−8^) (**Supplementary Figure 1**); however, both these mAb showed a slow off rate for rat C7 offering promise for use *in vivo*. Additionally, mAb 73D1 showed strong binding to mouse C7 (KD = 2.31 × 10^−9^) with a very slow off rate, promising for testing in mouse models. Analysis by SPR of immobilized 2H2 showed no measurable binding to native human, rat or mouse C7 in multiple analyses (negative data not shown).

**Table 2:** SPR analysis of the binding of C7 to the immobilized mAb.

Antibody	KD (M)	Ka (1/Ms)	Kd (1/s)
**17E7**	Human; 1.02 × 10^−9^	6.83 × 10^4^	6.94 × 10^−5^
**59E7**	Human; 9.31 × 10^−10^	1.45 × 10^6^	1.4 × 10^−3^
**3B11** (protein L captured)	Human; 2.30 × 10^−7^ Rat; 1.93 × 10^−7^	5.05 × 10^3^ 2.91 × 10^3^	1.20 × 10^−3^ 5.61 × 10^−4^
**73D1**	Human; 5.55 × 10^−8^ Rat; 8.17 × 10^−8^ Mouse; 2.31 × 10^−9^	2.63 × 10^4^ 6.98 × 10^3^ 1.02 × 10^5^	1.5 × 10^−3^ 5.70 × 10^−4^ 2.36 × 10^−4^

### The Novel mAbs Are Efficient Complement Inhibitors *In Vivo*

**Figure 4:**
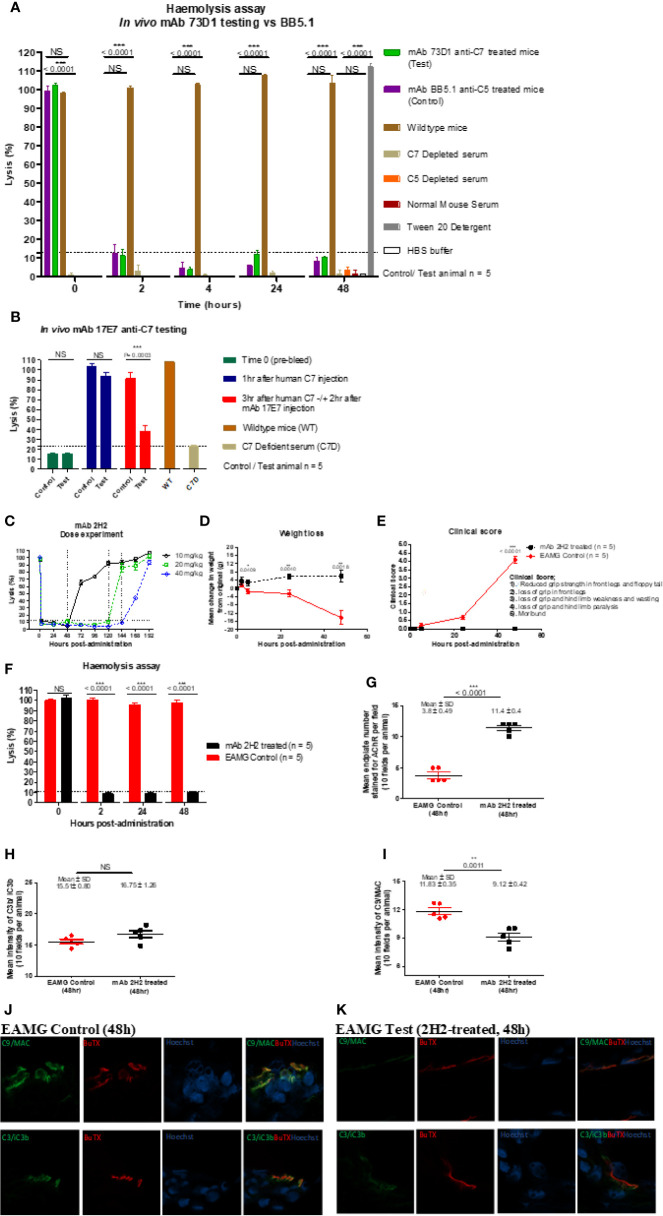
Testing anti-C7 mAbs *in vivo*. **(A)** mAb 73D1 or BB5.1 anti-mouse C5 as positive control was administered IP at a dose of 1 mg/kg to female wildtype mice (n=5 per group); blood was sampled at intervals serum obtained and added to human C7D or C5D serum respectively prior to measuring CP hemolytic activity measured. Controls included C7D and C5D human sera at the same dose, NMS to demonstrate the requirement for human depleted sera and 1% Tween-20 and HBS to set 100% and 0% lysis in the assay. Significance of differences between groups was determined by one-way ANOVA; significant differences and p values are shown in the figure. Error bars are standard errors of triplicates. **(B)** C7-deficient mice (10 females) were reconstituted with human C7 (500µg; IP), then split into test and control groups (5 in each). After 1 h, test and control animals were injected subcutaneously (SC) with 17E7 mAb or irrelevant isotype control mAb (1 mg) respectively. Blood was collected prior to administration of C7, immediately prior to administration of mAb and 3 h after mAb administration. Hemolytic activity was measured as above. Significance of differences between groups was determined using an unpaired t test; significant differences and p values are shown in the figure. Error bars are standard errors of triplicates. **(C)** Female Lewis rats were divided into three groups (n = 2 each) and injected intraperitoneally with mAb 2H2 at doses of 10, 20, and 40 mg/kg; blood was collected from all the animals just before mAb administration, 2 h after mAb 2H2 administration, and then every 12 h over 1 week. Sera prepared and hemolytic activity tested in standard CP assays. Significance of differences between groups was determined using an unpaired t test; significant differences and p values are shown in the figure. Error bars are standard errors of triplicates. **(D–F)** EAMG was induced in rats (5 per group) and either mAb 2H2 or isotype control mAb(10mg/kg) administered at induction. Weight loss **(D)** and clinical score **(E)** were measured at intervals; mice were bled at 0, 2, 24, and 48 h, serum harvested and hemolytic activity measured **(F)**. All animals were killed at 48 h. Results are means of five in each group and vertical bars represent SD. Significance of differences between groups was determined using an unpaired *t* test except panel E where paired t test was used; significant differences and p values are shown in the figures. **(G–K).** Soleus muscles were harvested at time of sacrifice (48 h) and snap frozen in OCT. Sections (10 µm) were stained for AChR with TRITC-conjugated a-BuTX; AChR-positive endplates were counted in 10 fields from each animal using ImageJ software. **(G)**. Sections were stained for C3b/iC3b **(H)** and C9/MAC **(I)** and staining quantified as above. Tissue sections from isotype control **(J)** or 2H2 **(K)** treated animals were double-stained for AChR together with anti-C3b/iC3b (top panel) or C9/MAC (bottom panel) and imaged on a Zeiss confocal microscope. The scale bar shown is 10 µm; all images were captured at identical magnification. Statistical significance was obtained by unpaired t-test and P < 0.05 was considered significant; significant differences and p values, mean and SD are shown in the figures.

The capacity of mAbs to inhibit complement *in vivo* was tested in mice and rats. To test the capacity of the anti-mouse C7 mAb 73D1 to inhibit complement *in vivo*, WT mice were administered 73D1 (or BB5.1 anti-mouse C5 as positive control) IP and complement activity in serum was tested at intervals over a time course of 48 h. Complement was inhibited by both mAb over the full course of the experiment up to end-point at 48 h (**Figure 4A**). To test the capacity of the anti-human C7 mAb 17E7 to inhibit C7 *in vivo*, mAb was administered to C7-deficient mice reconstituted with human C7. Human C7 effectively restored hemolytic activity in the mice, and administration of mAb 17E7 efficiently inhibited hemolytic activity in the mice (>70% inhibited at 3 h post-administration) compared to irrelevant antibody, demonstrating that the mAb blocked human C7 *in vivo* (**Figure 4B**). The rat-selective mAb 2H2 was tested for complement inhibition in rats to determine dose requirement and antibody half-life. Rats were injected with mAb 2H2 at 10, 20, and 40 mg/kg and blood collected at intervals for testing hemolytic activity. Even at the lowest dose, the mAb was an effective inhibitor, blocking complement activity for >48 h, and at the highest dose blocked complement for at least a week (**Figure 4C**). The 10-mg/kg dose was used for the EAMG experiment described below.

#### The Rat-Selective mAb 2H2 Prevents Clinical Disease and Pathology in a Myasthenia Model

EAMG was passively induced in rats; at the time of disease induction, the rat-selective mAb 2H2 or isotype control (10 mg/kg; 5 per group) was administered SC. As expected, all isotype control treated rats began to lose weight and show symptoms, comprising limp tails, piloerection, hind limb weakness and reduced grip strength within 24 h; all had a clinical score of 4 by endpoint (**Figures 4D, E**). (15–17) In contrast, animals given mAb 2H2 subcutaneously at the time of disease induction continued to gain weight over the time course of the experiment and did not develop detectable weakness or other clinical manifestations for the duration of the experiment (**Figures 4D, E**). Animals were sacrificed by a Schedule 1 method when weight loss was equal to or exceeded 20% of original bodyweight, when clinical score reached 4, or at the 48 h endpoint. CP hemolytic activity in serum was absent in the 2H2-treated group throughout the experiment (**Figure 4F**). As expected, serum from the untreated control animals retained full hemolytic activity across the time course. Soleus muscles were harvested at sacrifice, stained with α-Bungarotoxin-TRITC and receptor numbers quantified. The number of endplates in isotype control treated animals was significantly lower than in mAb 2H2-treated animals (~three-fold less; P < 0.0001, **Figure 4G**); endplate numbers in the 2H2-treated rat were comparable to numbers in naïve animals (data not shown). Endplates in muscles from the EAMG group were frequently fragmented, whereas most endplates in the 2H2-treated group were intact and linear, as in naïve animals. There was no significant difference in C3 fragment staining between the 2H2-treated and isotype control-treated animals (P = 0.9792, **Figure 4H**); however, the intensity of C9/MAC staining was reduced more than threefold in the 2H2-treated group (P = 0.0011, **Figure 4I**). Confocal analysis demonstrated co-localization of C3b/iC3b and C9/MAC deposition at the endplate in isotype control animals at 48 h (**Figure 4J**); in 2H2-treated animals, C3b/iC3b was deposited to a similar degree at endplates but C9/MAC deposition was weak or absent (**Figure 4K**).

## Discussion

The pathological role of complement in diverse diseases has been apparent for more than 50 years (20–23). Despite this long history, to date the use of anti-complement drugs has been restricted to a handful of rare diseases, including hemolytic disorders such as PNH and renal diseases, notably aHUS, where they have had a transformational impact (1–3). Eculizumab was first approved for treatment of PNH thirteen years ago, but only very recently have new anti-complement drugs reached the market and disease targets remain restricted to rare conditions (1). Recent reports provide irrefutable evidence that complement drives pathology in more common diseases including multiple sclerosis, NMOSD and AMD (24–26); however, because of the cost and route of administration, use of eculizumab in these diseases is not feasible; more affordable and effective drugs are needed. Complement proteins are abundant in plasma (~5% of total plasma protein), and the majority are acute phase reactants, increasing synthesis and plasma concentration in response to infection. This is a particular issue for C5 blockade; breakthrough hemolysis is a recognized complication in patients on eculizumab, likely because even a tiny amount of free C5 (<0.1%) is sufficient to restore hemolytic activity (27–29). These issues led us to consider other targets in the terminal pathway; we focused on C7 for four reasons: i). Plasma concentrations of C7 are lower than those of C5 (~2-fold lower molar concentration); ii). Unlike the other terminal pathway components, C7 is not synthesized by hepatocytes and is not an acute phase reactant, hence levels of C7 are stable in acute phase conditions (30); iii). Blocking C7 is likely to be less of an infection hazard compared to C5 blockade because C5a-mediated neutrophil recruitment is unimpaired - indeed, the majority of patients with C7 deficiency are healthy, although at increased risk of Neisserial infections (31); iv). C7 has been neglected as a therapeutic target, in contrast to C5 where numerous Pharma companies are developing blocking mAb or other drugs.

To identify clones secreting blocking anti-C7 mAbs early in clone screening we used a high-throughput hemolysis assay; notably, C7-blocking mAb were rare events, only 7 inhibitory clones identified from 15,000 screened clones over 15 separate fusions. Although we did not test for non-blocking anti-C7 mAbs in all fusions, this was done in early fusions and C7-binding activity was detected in ~5% of clones screened. This contrasts markedly with our experience with C5 where ~10% of the anti-C5 mAb generated were strong function blockers (17). The mAb described here bound C7 in ELISA (**Figure 2A**) and inhibited lysis of ShEA in CP assays (**Figure 1**) with mAb 17E7 and 59E7 being the most efficient for human serum; these mAb gave similar inhibition profiles to the currently available therapeutic anti-C5 mAb eculizumab and crovalimab. The mAb were generated in C7-deficient mice and several showed strong cross-species activity. The mAb 2H2 was most effective for rat serum and 73D1 for mouse serum; mAb 3B11 efficiently inhibited both human and rat serum (**Table 1**). Inhibition by 17E7 and 2H2 was confirmed in a modified CP assay using near-physiological serum concentrations. The *in vitro* inhibition data was confirmed *in vivo* for the mouse C7-blocking mAb 73D1, and for the human C7-specific mAb 17E7, the latter tested in C7-deficient mice administered human C7. Mouse serum inhibition by 73D1 was equivalent to the anti-C5 blocker BB5.1 (**Figure 4A**), widely used in animal models (19) and catalyzing the enthusiasm for anti-C5 therapeutics; the efficient inhibition of mouse complement by mAb 73D1 make it a valuable tool for animal studies targeting MAC specifically without interfering with C5a generation.

Both of the human C7-specific mAbs, 17E7 and 59E7, showed strong and stable binding to human C7 in SPR (KD = 1.02 × 10^−9^, 9.31 × 10^−10^, respectively; **Table 2**). Strong binding of mAb 73D1 to mouse C7 was also confirmed by SPR analysis (KD = 2.31 × 10^−9^, **Table 2**). Epitope specificity was tested in ELISA; surprisingly, all of the antibodies reactive with a given species C7 gave no signal when used as a pair in sandwich ELISA, showing that they bound the same or overlapping epitopes on C7 (**Figure 2B**). All the mAb worked as detect or capture in sandwich ELISA in conjunction with a polyclonal anti-C7, demonstrating that all bound C7 in this context. These observations suggest that there is a dominant epitope or surface of the C7 molecule that is critical for function and the target for all selected mAb. We plan structural studies to identify this epitope as we recently described for the anti-C5 mAb BB5.1 (19).

The mAb 2H2 stood out from the other mAb generated; this rat-selective mAb was a highly efficient complement inhibitor *in vitro* inhibiting at a dose at least 10-fold lower than the other in-house blocking mAb; at this dose, C7 is in ~10-fold molar excess compared to the mAb. Although 2H2 recognized C7 protein immobilized on plastic in direct ELISA, when tested in SPR immobilized on the chip surface, this mAb did not capture either human or rat C7 flowed over the surface. *In vivo*, this mAb completely blocked serum lytic activity at a quarter of the dose routinely used in rodent models (10 mg/kg versus 40 mg/kg body weight) for other terminal pathway inhibitory mAb, including BB5.1, the in-house anti-rat C5 4G2, and the 73D1 anti-C7 mAb (18, 19). When administered at the standard dose, 2H2 effectively blocked complement for at least seven days in rats, compared to 48 h for the other mAb at the same dose. Rats treated with the lowest dose of mAb 2H2 showed complete loss of hemolytic activity and were protected from disease compared to controls in the EAMG model; treated rats did not lose weight or develop paralysis, endplates were protected from damage and MAC deposition at endplates was markedly reduced (**Figures 4D–K**).

Taken together, the above data demonstrating that mAb 2H2 binds denatured but not native C7 and blocks hemolytic activity *in vitro* and *in vivo* at much lower concentrations than other blocking mAb and when C7 is in molar excess, suggested that 2H2 has a different mechanism of action to the other blocking mAb. We reasoned that it might inhibit by binding the transient C5b-7 complex. To test this, we used reactive lysis assays; all the blocking mAb, including 2H2, inhibited lysis when added to C5b6-bearing cells prior to addition of C7, indicating that they either prevented binding of C7 to C5b6 or its unfolding to reveal C8-binding sites. When the blocking mAb were added to preformed C5b-7 cells, only 2H2 inhibited suggesting that it additionally bound to C5b-7 and blocked C8 binding (**Figures 1G, H**). It was not possible to test directly the impact of 2H2 on the fluid-phase C5b-7 complex in these assays because of the transience of C5b-7 membrane-binding activity; instead, we explored binding of 2H2 and the other blocking mAb to forming and formed TCC in the fluid phase. Pull-downs from rat serum activated in the presence of mAb 2H2 included all terminal pathway proteins C5b–C9, demonstrating that the mAb captures intermediates in the fluid phase while permitting binding of later components to generate a mAb-TCC complex (**Figures 3F, G**). The anti-human C7 mAb 17E7, 59E7, and 73D1, when incubated in human serum during activation as above, each pulled down all the TCC components demonstrating that they too bound C7 in the complex and did not interfere with fluid-phase TCC generation (**Figures 3H–J**). Binding of the novel mAbs to pre-formed TCC was confirmed by sandwich ELISA capturing the complex on the aE11 anti-C9 neo-specific antibody (**Figures 2C, D**).

In some respects, the mechanism of action of mAb 2H2 resembles that of the naturally occurring MAC inhibitor clusterin; this fluid phase regulator binds C5b-7, preventing membrane attachment and insertion of the complex, but allows recruitment of C8 and C9 to form the TCC (32–34). Clusterin is a weak inhibitor of hemolysis that “buffers” bystander effects. The Streptococcal inhibitor of complement (SIC) protein similarly binds fluid-phase intermediates (predominantly C5b-7) and blocks the capacity of the complex to associate with membranes (35, 36). In contrast to 2H2, neither clusterin nor SIC caused inhibition in reactive lysis when C5b-7 was already on the target surface, confirming that they only interfere with membrane binding of nascent C5b-7. The capacity of 2H2 to bind and inhibit membrane-bound C5b-7 complexes suggests a dual impact on C5b-9 assembly; these properties make mAb 2H2 a powerful MAC inhibitor, a “super-clusterin” working with much greater efficiency to inhibit MAC assembly and resultant lysis.

In summary, we have demonstrated that, with regard to blocking hemolytic activity *in vivo* and *in vitro*, targeting MAC downstream of C5 is at least as effective as targeting C5. We describe anti-C7 mAbs that are powerful blockers of human, primate, rat and mouse C7, several working across species. We describe one mAb, 2H2, that shows selectivity for the C5b-7 complex and inhibits at greatly reduced dose compared to component-specific mAbs; although this mAb is predominantly active in rat, it is proof of principle that complex-specific mAb can be generated. These have potentially great advantages over current anti-complement drugs in terms of dose, frequency of administration and infection risk; they have the potential to open up new therapeutic fields for anti-complement drugs with significantly lower cost of treatment and more suited to treatment of common, chronic diseases. Current efforts are focused on the development of 2H2-like mAb that bind and block the human C5b-7 complex.

## Data Availability Statement

The raw data supporting the conclusions of this article will be made available by the authors, without undue reservation.

## Ethics Statement

The animal study was reviewed and approved by UK Home Office; license numbers: PF4167C0A and P8159A562.

## Author Contributions

WZ performed all the laboratory analyses and wrote the first draft of the manuscript. BPM conceived and planned the study and oversaw the data handling and manuscript preparation. All authors contributed to the article and approved the submitted version.

## Funding

WZ was supported by a PhD Studentship from the Life Sciences Research Network Wales (LSRN), LSRN Translational Support Fund, and by a Consolidator Award supported by Wellcome Trust ISSF funding to Cardiff University. BPM is supported by the UK Dementia Research Institute Cardiff.

## Conflict of Interest

BPM has provided advice on complement to Roche and is a consultant to RaPharma. The authors are named inventors on a patent (PCT/EP2020/073430) describing the anti-C7 mAbs.

## Supplementary Material

Supplementary Figure 1 Binding sensorgrams of the novel mAbs to C7 protein. mAb 3B11 (IgM) was immobilized on a protein L Series S sensor chip (GE Healthcare #29-2051-38) and mAb 73D1 (IgG2a, κ) immobilized on a mouse IgG capture sensor chip (GE Healthcare, # BR-1008-38) at approximately 60 RU. Human, rat or mouse C7 was flowed in HEPES-buffered saline (HBS) in a dilution range of 66 to 4 nM (3B11) or 66 to 8 nM (73D1) and interactions with the immobilized mAbs were analyzed. Sensorgrams were collected and KDs calculated using the Langmuir 1: 1 binding model with RI values set to zero. Sensorgrams are shown with raw data in colored lines and fitted data in dotted lines (average of 3); all binding data and analyses are included in **Table 2**. The SPR analysis was performed in an automated manner using T200 Biacore Evaluation Software version 2 (GE Healthcare).
