# Supplementary figures and images for: Monoclonal Antibodies Capable of Inhibiting Complement Downstream of C5 in Multiple Species
Source: Front Immunol. 2020 Dec 10;11:612402. doi: 10.3389/fimmu.2020.612402 (PMC7793867; doi:10.3389/fimmu.2020.612402)

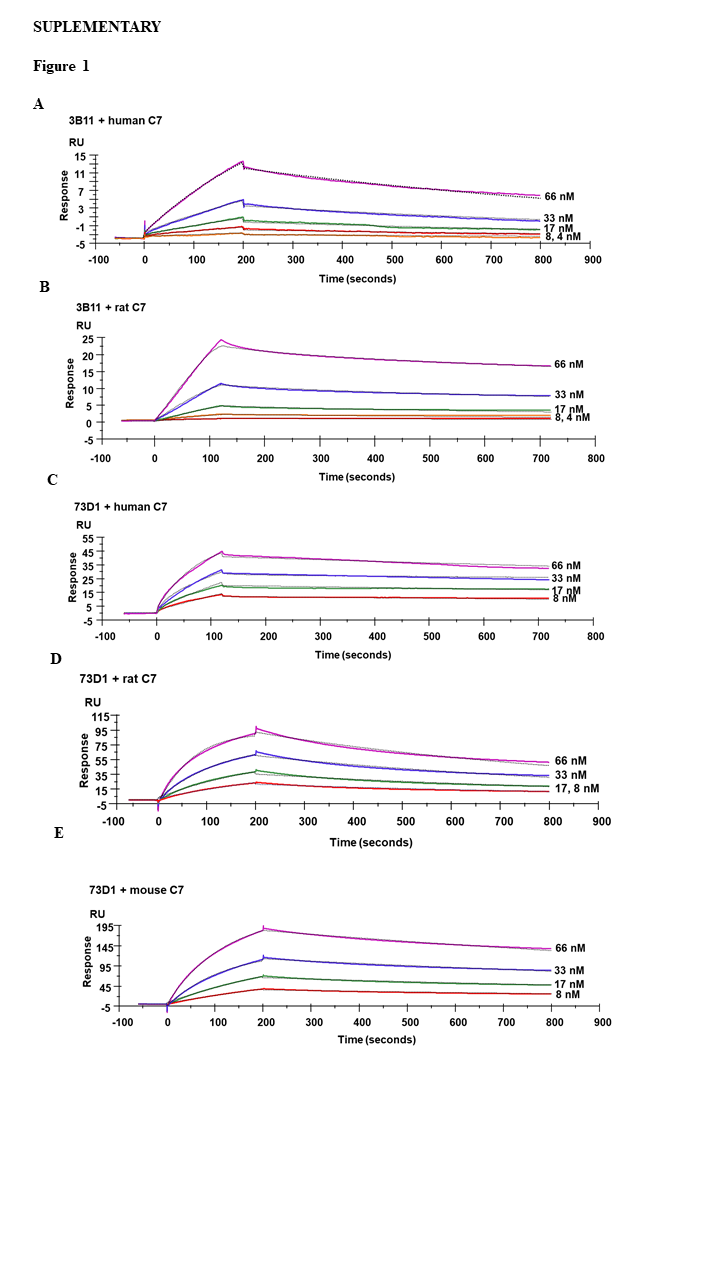

Supplement: Supplementary Figure 1 — Binding sensorgrams of the novel mAbs to C7 protein. mAb 3B11 (IgM) was immobilized on a protein L Series S sensor chip (GE Healthcare #29-2051-38) and mAb 73D1 (IgG2a, κ) immobilized on a mouse IgG capture sensor chip (GE Healthcare, # BR-1008-38) at approximately 60 RU. Human, rat or mouse C7 was flowed in HEPES-buffered saline (HBS) in a dilution range of 66 to 4 nM (3B11) or 66 to 8 nM (73D1) and interactions with the immobilized mAbs were analyzed. Sensorgrams were collected and KDs calculated using the Langmuir 1: 1 binding model with RI values set to zero. Sensorgrams are shown with raw data in colored lines and fitted data in dotted lines (average of 3); all binding data and analyses are included in Table 2 . The SPR analysis was performed in an automated manner using T200 Biacore Evaluation Software version 2 (GE Healthcare). [file Image_1.tif]
